# Attitudes Toward COVID-19 Vaccination Among Young Adults in Zurich, Switzerland, September 2020

**DOI:** 10.3389/ijph.2021.643486

**Published:** 2021-05-06

**Authors:** Cesar Leos-Toro, Denis Ribeaud, Laura Bechtiger, Annekatrin Steinhoff, Amy Nivette, Aja L. Murray, Urs Hepp, Boris B. Quednow, Manuel P. Eisner, Lilly Shanahan

**Affiliations:** ^1^ Jacobs Center for Productive Youth Development, University of Zurich, Zurich, Switzerland; ^2^ Department of Sociology, Utrecht University, Utrecht, Netherlands; ^3^ Department of Psychology, School of Philosophy, Psychology and Language Sciences, University of Edinburgh, Edinburgh, United Kingdom; ^4^ Integrated Psychiatric Services Winterthur-Zurcher Unterland, Winterthur, Switzerland; ^5^ Experimental and Clinical Pharmacopsychology, Department of Psychiatry, Psychotherapy and Psychosomatics, Psychiatric Hospital of the University of Zurich, Zurich, Switzerland; ^6^ Neuroscience Center Zurich, University of Zurich and Swiss Federal Institute of Technology, Zurich, Switzerland; ^7^ Institute of Criminology, University of Cambridge, Cambridge, United Kingdom; ^8^ Department of Psychology, University of Zurich, Zurich, Switzerland

**Keywords:** vaccine willingness, COVID-19, pandemic health communication, evidence-based health messaging, vaccine acceptance, vaccine hesitancy

## Abstract

**Objectives:** Young adults are essential to the effective mitigation of the novel coronavirus (SARS-CoV-2/COVID-19) given their tendency toward greater frequency of social interactions. Little is known about vaccine willingness during pandemics in European populations. This study examined young people’s attitudes toward COVID-19 vaccines in Fall 2020.

**Methods:** Data came from an ongoing longitudinal study’s online COVID-19-focused supplement among young adults aged 22 in Zurich, Switzerland (*N* = 499) in September 2020. Logistic regressions examined young adults’ likelihood of participating in COVID-19 immunization programs.

**Results:** Approximately half of respondents reported being unlikely to get vaccinated against COVID-19. Compared to males, females were more likely to oppose COVID-19 vaccination (*p* < 0.05). In multivariate models, Sri Lankan maternal background and higher socioeconomic status were associated with a greater likelihood of getting vaccinated against COVID-19 (*p* < 0.05). Respondents were more likely to report a willingness to get vaccinated against COVID-19 when they perceived 1) an effective government response (*p <* 0.05) and 2) their information sources to be objective (*p* < 0.05).

**Conclusion:** This study communicates aspects important to the development of targeted information campaigns to promote engagement in COVID-19 immunization efforts.

## Introduction

COVID-19, the disease caused by severe acute respiratory virus 2 (SARS-CoV-2), has demonstrated capabilities of rapid transmission across populations [1]. First reported in 2019, more than 2.4 million deaths and upward of 117 million cases have been attributed to the virus as of March 2021 worldwide [2]. There have been extensive challenges in responding to the COVID-19 pandemic due to a lack of knowledge of the natural history of the disease profiles it produces as well as rampant misinformation, skepticism and conspiracies related to the virus and its mitigation efforts [3, 4].

In November 2020, Switzerland had among the highest laboratory-confirmed COVID-19 cases per capita in the world [5]. Young people aged 20 to 29 in Switzerland had the highest infection rates across all age groups, posing a considerable threat to vulnerable populations [5]. Although younger people are less likely to experience serious negative health outcomes as a result of a COVID-19 infection, their tendency toward greater frequency of social interactions made them an essential group to consider in controlling the spread of the virus [6–10]. In December 2020, vaccine variants started to become available. As of March 2021, Switzerland had not completed vaccinations of the eldest and most at-risk groups of the population or published strategies to vaccinate the rest of the population, including young people [11]. Thus, it is urgent to better understand vaccine hesitancy among young adults as they are important disease vectors to address in order to mount an effective immunization campaign [12–15]. It remains unclear whether COVID-19 vaccination limits further transmission, however, it is understood to protect vulnerable populations. According to the Strategic Advisory Group of Experts (SAGE) on Immunization, vaccine hesitancy is a behavior influenced by a number of factors including issues of confidence (trust in vaccine provider), complacency (perceptions of need and/or value of a vaccine), and convenience (access) [16].

Switzerland has among the lowest levels of vaccine confidence internationally [17]. In Switzerland, vaccinations are not compulsory and a national vaccination registry does not exist [18, 19]. This strategy was designed to provide autonomy in local governance but has led to suboptimal vaccine coverage among the general population [18, 20]. Coverage between cantons is variable, for example, measles vaccination coverage is lower in German- (71.3%) than French-speaking (85.0%) cantons [21]. Similarly, individuals in French-speaking cantons report greater influenza vaccine coverage than those in German-speaking cantons across all age groups [22]. Vaccine hesitancy and refusal leads to under-immunization, reduction in potential for reaching herd immunity, and increases the risk of preventable illness and mortality. The success of mitigating outbreaks is dependent on the public’s willingness to engage with preventive public health programs including immunization.

Reasons for accepting or declining preventive measures in pandemic situations include uncertainties of the severity of a disease agent, perceptions that vaccines are rushed to market, among others [23–27]. Emerging work on COVID-19 vaccine acceptance demonstrates that these attitudes are highly variable worldwide. Among European populations, approximately 7 in 10 (73.9%) reported a willingness to get vaccinated against COVID-19 in early April 2020 [28]. Over time, vaccine acceptance has been reported to be as low as 53.7% in Italy and as high as 80% in Denmark according to more recent data [27, 29, 30]. Decreases in vaccine acceptance were observed in Europe throughout the pandemic which is thought to be associated with lower perceived confidence in vaccine safety and effectiveness among populations [29]. Public perceptions of vaccines in Switzerland are poorly understood; existing research suggests a great influence of local socio-cultural contexts as determinants of vaccination decisions which may be distinct by Swiss region [31, 32]. More recent public opinion polls and peer-reviewed literature from around the world have noted a decreased willingness. For example, among adults in the United Kingdom and United States, approximately half responded that they would likely get vaccinated; in Switzerland, only 16% had the same response [3, 33–35]. These attitudes are not fixed and may change as nations report on successes.

Young people form risk perceptions related to health issues, in part, based on their perceptions of susceptibility and seriousness of a particular disease as well as social patterning from respected peers’ behavior [36]. Higher participation in immunization programs may occur when individuals believe that effective protections exist and when they are directed or enabled to engage in such activities [37]. Young people may base personal vaccine uptake decisions on misinformed or shortsighted heuristics whereby they perceive the side-effects related to a vaccine to be more likely than the disease it may prevent [38, 39]. These perceptions are informed by their physical and social environment which includes mass public health information campaigns. Successful campaigns create enabling conditions to seek reliable information on prevention and other services to help mitigate a diseased state. For example, human papilloma virus (HPV) vaccination rates among adolescent young Swiss women aged 16 years were 51% if they attended schools with a vaccination program and 37% if their school did not have such a program [39]. In Switzerland, there have been limited public attempts to actively engage and educate young people about their role in combatting the COVID-19 pandemic. The most high profile appeal for limiting social contact among young people came from the WHO in March 2020 [40]. It is unclear if and how this message was disseminated or tailored to young people in Switzerland. In the absence of reliable information campaigns, young adults may turn to the internet as a primary source of information about COVID-19 where there is little information quality control [41]. Scientific journals, media outlets and other respected sources of information were not immune to baseless claims during this pandemic, including conspiracy theories related to COVID-19 vaccine development and trials [42–48]. Perceptions of this media landscape and their associations with vaccine uptake are currently unknown among young people living in Switzerland in the context of the COVID-19 pandemic.

Sociodemographic characteristics have been observed to influence behavior and shape differences in cultural norms around vaccine uptake. For example, women tend to be more averse to vaccine uptake than males [49, 50]. Existing literature suggests differences in immune responses between the sexes, however, sex is not considered in the design or dosing of recommended immunizations, which may lead to undocumented adverse effects disproportionately affecting females [51]. Women also report more influenza vaccination concerns in terms of its efficacy and safety, and report more adverse reactions to vaccines in general which may explain their relatively lower uptake than men and greater hesitancy to participate in immunization programs [49, 50]. Higher education has also been associated with increased uptake of vaccines over the life course [52]. Differences in national background have been observed in vaccine willingness in Switzerland; non-Swiss children have higher vaccine coverage levels than Swiss children [53]. Relatively greater household economic resources has also been observed to affect vaccination attitudes and behaviors although this has been a confounder in some studies [32, 54].

To optimize COVID-19 vaccine utility, it is essential that national health authorities have an evidence base to inform the (re)allocation of resources to key and strategic subpopulations and to develop strategies to combat deficiencies in health literacy related to the COVID-19 pandemic. These activities should include the development of mass communication campaigns related to vaccination and clarifications of prevalent misinformation.

The current prospective, community-based study took place in Switzerland’s largest city, Zurich, over the course of the first 6 months of the COVID-19 outbreak in Europe and collected data specific to young people’s perceptions of the COVID-19 virus and their opinions on a hypothetical vaccine compliance strategy implemented by the Swiss government. The study sought to characterize its respondents along sociodemographic factors and provide perspectives important to the development of an evidence base for Swiss-specific pandemic responses targeting young people.

We expect that *H1:* there will be significant associations between female sex and lower vaccine willingness, *H2:* significant associations between perceptions of a favorable government response and media coverage and vaccine willingness, and that *H3:* experience with COVID-19 will be significantly associated with agreement to a mandatory COVID-19 vaccine.

## Methods

### Design

Data came from a supplement to the Zurich Project on the Social Development from Childhood to Adulthood (*z-proso*), an ongoing prospective longitudinal study on the development of prosocial and problem behavior (e.g., antisocial behavior) from childhood to adulthood. A total of eight waves of data, at ages 7, 8, 9, 11, 13, 15, 17, and 20, were collected from children who, in 2004, entered first grade in one of 56 public primary schools in Zurich. Stratified random sampling procedures used in the initial target sample of schools oversampled less resourced school districts. Details on original sample selection and attrition have been detailed elsewhere [55, 56]. Respondents who had participated in the age-20 assessment (2018, *N* = 1,180) were invited to participate in four data collections after Switzerland went into its first home semi-confinement period which were conducted in German: Supplement 1 (8–14 April 2020, *N* = 786), Supplement 2 (30 April–5 May 2020, *N* = 650), Supplement 3 (21–26 May 2020, *N* = 569), and Supplement 4 (10–15 September 2020, *N* = 525). The current analyses primarily use data from Supplement 4, when data on vaccination willingness was collected, as well as assessments from ages 13 and 15 when the most complete records for socioeconomic indicators were collected.

At ages 13 and 15, participants completed a paper questionnaire in classrooms outside of regular class times. At age 20, the questionnaire was conducted on a computer at a Decision Science Laboratory of the Swiss Federal Institute of Technology (ETH) in Zurich which typically lasted 90 min. At ages 13 and 15, respondents were compensated with 50 Swiss Francs for their time. Those who had participated in the age-20 *z-proso* data collection were invited by SMS and e-mail to participate in the supplementary COVID-19 data collection through a personalized link. Respondents of the *z-proso* COVID-19 Supplements, 1–4, were entered into a lottery with the opportunity to win one of 50 prizes of 100 Swiss Francs each. At each of the four Supplement waves, participants were given 7 days to complete the survey. The study was reviewed by and received ethics clearance from the Ethics Committee of the Faculty of Arts and Social Sciences of the University of Zurich.

### Measures

*Sociodemographic variables* included: sex (female, male), education level (compulsory school, vocational/technical training, higher education), and household International Socio-Economic Index of Occupational Status (ISEI) Quartile at age 13/15 [57]. Preliminary analyses of the data revealed no significant differences between respondents with Swiss or Northwestern European maternal backgrounds; therefore, we coded these participants into one Western European group. Thus, categories presented include: Western European, Southern European, Sri Lankan, Middle East and African, and American and other maternal background.

*Perceptions of Swiss authorities* ‘*response, politicians*’ *views, and media coverage* were assessed with the following question and statements: “What are your opinions and attitudes toward the measures against the coronavirus? How much truth do the following statements have for you?”; “The measures taken by the Swiss authorities to deal with the coronavirus are effective.”; “I agree with the measures taken by the Swiss authorities to combat the coronavirus”; “Politicians and the media exaggerate the threat”; “The media provides balanced and objective information about the coronavirus.” Answer options included: 1 = Not true at all, 2 = Not really true, 3 = Somewhat true, and 4 = Completely true; these were dichotomized to: 0 = Not true at all/not really true, and 1 = Somewhat true/completely true.

*Personal health experiences with COVID-19.* Exposure of a family member, partner, or other close person to COVID-19 was assessed with the probe, “A family member, partner or other close person…” where 1 = has tested positive for COVID-19, but did not have to go to the hospital because of it, 2 = is/was sick with COVID-19 and therefore had to go to the hospital, 3 = died as a result of the coronavirus illness. Each of these three items had answer options 1 = Yes, 2 = No. A positive indication to any of the three items probed across the four waves of data collection that occurred during the pandemic was coded as: 0 = Family member, partner or related person’s health not affected by COVID-19, alternatively 1 = Family member, partner or related person’s health affected by COVID-19 if there were positive responses to the probes. Next, a respondent’s personal health affected by the COVID-19 was assessed using the probe, “I myself…” where 1 = had symptoms that could be attributed to the coronavirus with answer options 1 = Yes, 2 = No. If respondents indicated a positive response to the probe in any of the four data collection waves that were conducted during the pandemic, they were coded as 1 = Personal health affected by COVID-19 pandemic, 0 = Personal health not affected by the COVID-19 pandemic. Initially, Swiss authorities established regulations that barred asymptomatic and subclinical suspected cases from testing which is the most common presentation of a COVID-19 infection among young people, thus, self-reported data may provide a better estimate of prevalence of disease among this population [58].

*Attitudes toward vaccination* were assessed with the following hypothetical: “Imagine that Switzerland has officially approved a vaccine against the coronavirus.” “Would you get vaccinated?”; answer options: 1 = Yes, for sure, 2 = Yes, probably, 3 = Undecided, 4 = No, probably not, and 5 = Definitely not. A binary variable was created to assess respondents’ reported *likelihood of getting vaccinated* coded as 1 = Likely to get vaccinated and 0 = Not likely to get vaccinated or undecided should a vaccine become available. Delving deeper, *undecided about vaccination* was coded as 1 = Undecided and 0 = Decided either way. Participants were also asked, “Would you agree to an obligatory vaccination if the public health authorities mandated it?” with answer options, 1 = Completely agree, 2 = Somewhat agree, 3 = Somewhat disagree, and 4 = Completely disagree. To assess whether respondents would be *agreeable to a mandatory vaccine*, a binary variable was created coded as 1 = Agree and 0 = Disagree.

### Analysis

All analyses were conducted using SPSS (Version 26.0, Armonk, NY: IBM Corp.). Survey weights were used to allow for generalization back to the original recruitment population (*see* Nivette et al. 2021). Logistic regression models examined whether personal and peripheral experiences with COVID-19 and perceptions of the Swiss government response and media portrayal of the pandemic were associated with attitudes toward vaccination including likelihood of vaccine uptake, being undecided about vaccination, and agreeableness to a compulsory vaccination. Final models included sex, maternal national background, respondents’ education level, household ISEI quartile, perceptions of governmental response, perceptions of media portrayal of the pandemic, and personal experiences with COVID-19. Sample weights were applied to all analyses.

## Results

**FIGURE 1:**
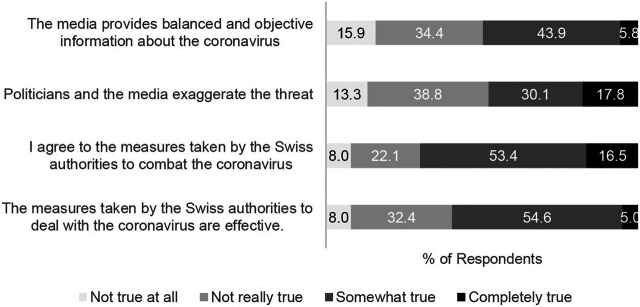
Perceptions of Swiss authorities ‘response, politicians’ views, and media coverage (*N* = 499); Zurich Project on the Social Development from Childhood to Adulthood, Zurich, Switzerland, September 2020.

Sample characteristics are displayed in Table 1. Overall, 525 respondents completed the fourth *z-proso* COVID-19 supplement survey which included the questions on vaccination. However, respondents who reported being outside of Switzerland at the time that the survey was administered were excluded; hence the analytic sample of 499. Perceptions of Swiss authorities’ response, politicians’ views, and media coverage are presented in Figure 1.

**TABLE 1:** Sample characteristics; Zurich Project on the Social Development from Childhood to Adulthood, Zurich, Switzerland, September 2020.

Sample characteristics		Unweighted (*N* = 498)		Weighted (N = 499)
		*n*	%	%
Sex	Female	300	60.2	47.7
	Male	198	39.8	52.3
Maternal Country of Birth				
	Western Europe	303	60.8	52.6
	Southern Europe	69	13.9	21.7
	Sri Lanka	26	5.2	5.4
	Middle East and Africa	68	13.7	12.6
	American and Other	28	5.6	7.0
Highest Education Attained, Age 20				
	Compulsory School	50	10.0	10.4
	Vocational/Technical Training	273	54.8	58.0
	Higher Education	175	35.1	31.6
International Socio-economic Index of Occupational Strata				
	Lowest 25%	112	22.5	28.1
	Q2	127	25.5	24.7
	Q3	127	25.5	22.8
	Highest 25%	122	24.5	22.1
Perceptions of Swiss Government Response, Fall 2020				
	Effective	305	61.2	59.4
	Agrees with response	359	72.1	69.9
Perceptions of the Media’s and Politicians' Views of COVID-19, Fall 2020				
	Not exaggerated	271	54.4	52.0
Perceptions of Media Coverage, Fall 2020				
	Balanced and objective	245	49.2	49.6
Personal health experiences with COVID-19, April—September 2020				
Personal health and health of close person(s)^a^unaffected		211	42.4	42.5
Personal health and health of close person(s)^a^affected		90	18.1	18.4
Only health of close person(s)^a^affected		76	15.3	15.2
Only personal health affected		121	24.3	24.0

Note: Western Europe: Switzerland, Italy, Portugal, Spain, Germany, European Union 15 Member States/European Free Trade Association; Southern Europe: Serbia/Montenegro/Kosovo, Bosnia-Herzegovina, Croatia, Macedonia, South and East Europe; Middle East and Africa: North Africa, Sub-Saharan Africa, Near East, Middle and Far East, Turkey; Americas and Other: United States of America, Canada, New Zealand, Australia, Brazil, Other Latin America, Other.

a Close person(s): include family member, partner, or related person.

### Vaccination Willingness

Table 2 shows that approximately half (46.9%) of young people surveyed reported that they would likely get vaccinated if a vaccine became available. Final adjusted logistic regression analyses indicated that females were less likely than males to indicate vaccination willingness (*p* < 0.001, OR = 0.46, 95%CI = 0.30–0.71). Respondents with a Sri Lankan maternal background were more likely to report that they would get vaccinated than respondents of other cultural backgrounds (*p* < 0.01); this Sri Lankan maternal background became significant and stronger from the bivariate models (Table A1) to the adjusted models (in Table 2), with the adjustment of socioeconomic status. Thus, when SES is held equal, participants with a Sri Lankan background are more likely to report a higher likelihood of getting vaccinated. Lower educational attainment was associated with lower likelihood of vaccine willingness. Individuals with vocational or technical training were less likely to report vaccine willingness than those with higher education (*p* = 0.015, OR = 0.55, 95%CI = 0.34–0.89). Compared to respondents in the lowest ISEI quartile, all others were more likely to be willing to get vaccinated against COVID-19 (*p* < 0.01).

**TABLE 2:** Attitudes toward a COVID-19 vaccination among young people in; Zurich Project on the Social Development from Childhood to Adulthood, Zurich, Switzerland, September 2020.

Q. Imagine that Switzerland has officially approved a vaccination against the coronavirus			
	Unweighted (*n* = 496)		Weighted (*n* = 497)
	*N*	%	%
Would you get vaccinated?			
Yes, for sure	102	20.5	19.5
Yes, probably	140	28.1	27.4
Undecided	97	19.5	19.9
No, probably not	79	15.9	16.9
No, definitely not	78	15.7	16.0
	Unweighted (*n* = 495)		Weighted (*n* = 495)
Opinion on authorities making vaccine mandatory	*n*	%	%
Totally agree	83	16.7	17.0
Somewhat agree	145	29.1	28.9
Somewhat disagree	134	26.9	26.0
Disagree completely	133	26.7	27.5

Table 3 shows that respondents who perceived that the Swiss national response was effective and that politicians provided non-exaggerated views of the COVID-19 situation were more likely to report a greater likelihood of getting vaccinated than those who reported otherwise (*p* = 0.048, OR = 1.64, 95%CI = 1.00–2.68 and *p* < 0.001, OR = 3.93, 95%CI = 2.49–6.21 respectively). Finally, personal experience with COVID-19 was not significantly associated with greater likelihood of vaccination willingness. One exception was that respondents whose own health and the health of close ones was reportedly affected by COVID-19 were less likely to report vaccination willingness than when only they themselves had been affected (*p* = 0.026, OR = 0.49, 0.26–0.92).

**TABLE 3:** Multivariate logistic regression analyses examining likelihood of getting vaccinated, vaccine hesitancy, and opinion to compulsory vaccination, weighted analyses; Zurich Project on the Social Development from Childhood to Adulthood, Zurich, Switzerland, September 2020.

Characteristic	Ref. Category	Model 1			Model 2			Model 3		
		Likelihood of getting vaccinated			Undecided about vaccination			Agree to compulsory vaccination		
		*p*	OR	95%CI	*p*	OR	95%CI	*p*	OR	95%CI
Sex										
Female	Male	<0.001	0.46	0.30–0.71	0.039	1.66	1.03–2.67	0.002	0.52	0.35–0.79
Maternal Country of Birth										
Southern Europe	Western Europe	0.472	0.81	0.45–1.45	0.478	1.24	0.68–2.27	0.951	1.02	0.58–1.79
Sri Lanka		0.005	4.26	1.56–11.66	0.029	0.18	0.04–0.84	0.007	4.01	1.45–11.07
Middle East and Africa		0.913	0.96	0.49–1.90	0.422	0.74	0.36–1.54	0.458	1.27	0.67–2.41
Americas and Other		0.582	0.79	0.33–1.86	0.841	0.91	0.35–2.38	0.706	0.85	0.36–1.99
Southern Europe	Americas and Other	0.956	1.03	0.39–2.73	0.556	1.37	0.48–3.94	0.708	1.20	0.46–3.10
Sri Lanka		0.009	5.43	1.54–19.14	0.072	0.20	0.03–1.16	0.016	4.73	1.34–16.67
Middle East and Africa		0.693	1.23	0.44–3.40	0.723	0.82	0.27–2.49	0.418	1.50	0.56–4.00
Southern Europe	Sri Lanka	0.002	0.19	0.07–0.54	0.016	6.89	1.44–32.97	0.011	0.25	0.09–0.73
Middle East and Africa		0.008	0.23	0.08–0.68	0.084	4.10	0.83–20.36	0.039	0.32	0.11–0.95
Southern Europe	Middle East and Africa	0.656	0.84	0.38–1.83	0.211	1.68	0.75–3.78	0.549	0.80	0.38–1.66
Highest Education Attained, Age 20										
Vocational/Technical Training	Compulsory Schooling	0.829	0.93	0.46–1.87	0.467	1.37	0.59–3.15	0.901	1.05	0.53–2.08
Higher Education		0.173	1.69	0.79–3.60	0.377	1.51	0.61–3.75	0.218	1.59	0.76–3.35
Vocational/Technical Training	Higher Education	0.015	0.55	0.34–0.89	0.730	0.91	0.52–1.59	0.082	0.66	0.41–1.06
International Socio-economic Index of Occupational Strata										
Q2	Lowest 25%	0.033	1.93	1.06–3.53	0.067	0.56	0.31–1.04	0.205	0.69	0.39–1.22
Q3		0.007	2.39	1.27–4.52	0.011	0.41	0.21–0.82	0.758	0.91	0.50–1.66
Highest 25%		0.003	2.76	1.40–5.44	0.017	0.40	0.19–0.85	0.262	1.45	0.76–2.76
Q2	Highest 25%	0.255	0.70	0.38–1.29	0.351	1.41	0.69–2.89	0.016	0.48	0.26–0.87
Q3		0.637	0.87	0.48–1.56	0.942	1.03	0.51–2.07	0.107	0.63	0.36–1.11
Q3	Q2	0.464	1.24	0.70–2.20	0.346	0.73	0.38–1.41	0.341	1.32	0.75–2.32
Perceptions of Swiss Government Response to COVID-19 pandemic, Fall 2020										
Effective	Not effective	0.048	1.64	1.00–2.68	0.021	0.53	0.31–0.91	0.400	1.23	0.76–1.97
Agrees to measures taken	Does not agree	0.795	1.07	0.63–1.85	0.083	1.70	0.93–3.10	0.317	1.31	0.77–2.22
Perceptions of Media and Politicians’ views of COVID-19, Fall 2020										
Non-exaggerated views	Exaggerated views	<0.001	3.93	2.49–6.21	0.306	0.77	0.46–1.28	<0.001	2.59	1.68–3.99
Perceptions of Media Coverage, Fall 2020										
Fair and Balanced	Not fair or balanced	0.062	1.53	0.98–2.38	0.887	0.96	0.58–1.60	0.001	2.01	1.32–3.08
Personal health experiences with COVID-19, April—September 2020										
Personal health and health of close person(s) affected	Personal health and close person(s) unaffected	0.258	0.72	0.41–1.27	0.162	1.54	0.84–2.90	0.479	1.22	0.70–2.11
Only health of close person(s) affected		0.621	1.17	0.63–2.15	0.993	1.00	0.51–1.96	0.074	1.71	0.95–3.06
Only personal health affected		0.142	1.48	0.88–2.50	0.754	1.10	0.61–1.98	0.331	1.28	0.78–2.13
Personal health and health of close person(s) affected	Only personal health affected	0.026	0.49	0.26–0.92	0.336	1.40	0.71–2.76	0.869	0.95	0.52–1.75
Only health of close person(s) affected		0.483	0.79	0.40–1.54	0.799	0.91	0.43–1.91	0.385	1.33	0.70–2.52
Only health of close person(s) affected	Personal health and health of close person(s) affected	0.621	1.17	0.63–2.15	0.993	1.00	0.51–1.96	0.074	1.71	0.95–3.06

Note: Western Europe: Switzerland, Italy, Portugal, Spain, Germany, European Union 15 Member States/European Free Trade Association; Southern Europe: Serbia/Montenegro/Kosovo, Bosnia-Herzegovina, Croatia, Macedonia, South and East Europe; Middle East and Africa: North Africa, Sub-Saharan Africa, Near East, Middle and Far East, Turkey; Americas and Other: United States of America, Canada, New Zealand, Australia, Brazil, Other Latin America, Other.

a Close person(s): include family member, partner, or related person.

### Undecided About Vaccination

A significant minority of respondents, approximately 1 in 5 (19.9%), reported being undecided about getting vaccinated. Table 3 shows that female respondents were more likely to be undecided than males (*p* = 0.039, OR = 1.66, 95%CI = 1.03–2.67). In final adjusted models, Sri Lankans were less likely than those with Western European backgrounds to report being undecided about vaccination (*p* = 0.029, OR = 0.18, 95%CI = 0.04–0.84). Conversely, Southern Europeans were much more likely to report being undecided than those with Sri Lankan backgrounds (*p* = 0.016, OR = 6.89, 95%CI = 1.44–32.97). Respondents in the top two socioeconomic quartiles (Q3 and Q4) were less likely to be undecided than those in the lowest quartile (*p =* 0.011, OR = 0.41, 95%CI = 0.21–0.82 and *p* = 0.017, OR = 0.40, 95%CI = 0.19–0.85 respectively). Finally, those who believed that the Swiss government had responded effectively were less likely to be undecided (*p* = 0.021, OR = 0.53, 95%CI = 0.31–0.91).

### Agreement to Compulsory Vaccination

When asked about their opinion on a situation whereby the authorities implemented a compulsory vaccination mandate, approximately half (45.9%) of young people surveyed were agreeable to this strategy. However, female respondents were less likely to agree than males (*p* = 0.002, OR = 0.52, 95%CI = 0.35–0.79). Respondents with a maternal Sri Lankan background were more likely to agree than those who identified any other national background (*p* < 0.05). Differences in responses by ISEI were limited to those in Q_2_ being less likely than those in the highest ISEI quartile to agree to such a mandate (*p* = 0.016, OR = 0.48, 95%CI = 0.26–0.87). Finally, when respondents perceived that politicians provided non-exaggerated views and that the media provided fair and balanced coverage of the pandemic, they were more likely to agree to compulsory, health authority-mandated vaccinations (*p* < 0.001, OR = 2.59, 95%CI = 1.68–3.99 and *p* = 0.001, OR = 2.01, 95%CI = 1.32–3.08).

## Discussion

In September 2020, before any COVID-19 vaccine was approved worldwide, almost half of young adults in Zurich were either undecided or unlikely to get vaccinated against COVID-19 and also would not agree with coercive vaccine mandates. This is generally consistent with recent Swiss general population surveys, which reported that COVID-19 vaccine willingness has eroded as the pandemic and social restrictions have been prolonged [34, 59]. These low rates of vaccine willingness among youth and young adults is concerning; their cooperation with mitigation efforts such as vaccinations are essential to combatting COVID-19 at a national level [5]. To date, no information campaigns have yet been tailored to or systematically deployed specifically to young people in Swiss settings that expound on their importance to COVID-19 eradication efforts and the collective interest in their participation in immunization programs.

Historically, vaccination rates in Switzerland have not always been high enough to protect the population (18). Arguably, parts of the population do not sufficiently endorse vaccines as an acceptable and effective strategy to prevent disease or mortality. Coercive immunization programs in certain countries have proven to be effective in reducing morbidity and mortality of vaccine preventable diseases, however, they are ethically fraught, and may lead to further resistance [60, 61]. Switzerland values plurality and policy diversity among its different cantons; this is reflected in the cantons’ different immunization programs and outcomes. Deficiencies in vaccine compliance among cantons have been attributed to differing priorities and practices of policy makers and attitudes of vaccine gate-keepers (e.g., nurses, general practitioners) and engagement in alternative health care and education pedagogies [32, 53, 62]. Swiss communities report an openness to public programs if their autonomous decision-making is respected [62–66].

The findings of the current study suggest that in Fall 2020, young women in Switzerland were less willing to get vaccinated against COVID-19, more undecided about vaccination, and unlikely to agree to a mandatory vaccine. These findings are consistent with existing research on sex differences in vaccine acceptance and uptake.

In final adjusted models, Sri Lankan maternal background was associated with greater vaccination willingness, lower vaccine undecidedness, and more agreement to mandated immunization than all other national groups in the sample. These findings only emerged when socioeconomic background, which is a considerable social determinant of health and health behaviors, was adjusted for. Sri Lanka has vaccine coverage approaching 99% for most vaccine-preventable diseases [67]; thus, young adults with a Sri Lankan background may have stronger cultural norms of participating in vaccination programs compared to young adults from other nationalities with comparable socioeconomic status [68].

Young adults in the current study in higher ISEI quartiles were more likely to report vaccine willingness and being decided about vaccination. In other studies, socioeconomic status has been a confounder to vaccine willingness, as it has been associated with greater likelihood of an acceptance of alternative medicines that are not based on a biomedical model of health [69]. Future studies should continue to untangle the mechanisms that drive these alternative attitudes in order to identify intervention points that optimize local public health strategy.

Finally, the current study provides concrete evidence that while the population of young adults in Switzerland does not necessarily have to agree with the extraordinary measures that the Swiss government took in order to perceive that the national response was effective. When presented with what young people perceive to be balanced views from politicians, decision makers, and media, young adults are more open to participating in immunization programs and become more open to paternalistic strategies to mitigate the spread of COVID-19. These findings provide an evidence base on which to build mass information campaigns dedicated to subpopulations of young adults that adequately inform and build resilience against misinformation that may otherwise dissuade them from immunization efforts. Furthermore, it identifies women, those with relatively lower educational achievement, and socioeconomically disadvantaged groups as priorities for immunization information campaigns. Any public health effort should tailor messaging that is relevant to the target group, contains clear guidelines, and is easily understandable to improve health literacy specific to COVID-19 or any other contagion. When information is too complex, it fails to lead to disambiguation, increases feelings of despair and panic, and further confuses an already stressed population [70].

The current study used a broad and diverse sample that is representative of young adults living in Zurich during the time of the COVID-19 pandemic. While it may not be representative of French- or Italian-speaking Switzerland, it provides a measure that may be standardized that informs willingness to participate in vaccination programs. The findings may be useful during times of epidemics but also generally inform the Swiss National Vaccination Strategy and related public health activities. The substantial attrition across survey waves is a limitation mitigated with the development and application of survey weights to reflect the original sample in all analyses; it may have been further mitigated with a longer data collection period. The original target sample was generally representative of first-graders in Zurich in 2004. In the current study, we used sampling weights that allowed us to generalize findings back to this original sample, which consisted of a balanced sample of males and females. The use of non-validated scales is a potential limitation, but also a necessity during times of novel stressors for which we may not have well-established scales. The current study identifies groups that may have strong vaccine-adherence practices based on their national background that should be leveraged to inform public health practice in Swiss settings—specifically, factors that sustain and maintain strong immunization compliance. Furthermore, we emphasize the importance of including sex-based considerations in vaccine messaging development and identify populations that may be at risk of future immunization deficiencies.

Overall, our findings suggest that approximately half of young people living in Switzerland’s largest city report being unlikely to participate in COVID-19 immunization programs. However, when young adults perceived information in their media landscape to be balanced and objective, they were more likely to report a willingness to get vaccinated and even participate in a health authority-mandated vaccination program. In order to create and sustain these attitudes, targeted public information campaigns relatable to young adults, delivered at an appropriate language level that are easy to understand are essential to creating enabling conditions for participation in improving immunization coverage efforts.

## Figures and Tables

**TABLE A1:** Bivariate logistic regression analyses examining likelihood of getting vaccinated, vaccine hesitancy, and opinion to compulsory vaccination, weighted analyse; Zurich Project on the Social Development from Childhood to Adulthood, Zurich, Switzerland, September 2020.

	Likelihood of getting vaccinated					Likelihood of being undecided about getting vaccinated				Likely to agree to compulsory vaccine			
	Ref.	*p*	OR	95% CI		*p*	OR	95% CI		*p*	OR	95%CI	
Sex													
Female	Male	0.008	0.63	0.44	0.88	0.136	1.39	0.90	2.16	0.003	0.59	0.42	0.83
Maternal Country of Birth													
Southern Europe	Western Europe	0.008	0.53	0.33	0.85	0.203	1.43	0.82	2.48	0.254	0.76	0.48	1.22
Sri Lanka		0.451	1.37	0.60	3.12	0.357	0.56	0.16	1.93	0.043	2.43	1.03	5.76
Middle East and Africa		0.012	0.50	0.29	0.86	0.750	1.11	0.58	2.14	0.418	0.80	0.47	1.37
Americas and Other		0.040	0.45	0.21	0.96	0.982	0.99	0.39	2.51	0.144	0.57	0.26	1.21
Southern Europe	Americas and Other	0.711	1.17	0.51	2.72	0.473	1.44	0.53	3.95	0.491	1.34	0.58	3.11
Sri Lanka		0.042	3.06	1.04	8.95	0.455	0.57	0.13	2.52	0.010	4.30	1.42	13.00
Middle East and Africa		0.816	1.11	0.46	2.68	0.830	1.12	0.39	3.26	0.432	1.42	0.59	3.40
Southern Europe	Sri Lanka	0.036	0.38	0.16	0.94	0.155	2.56	0.70	9.31	0.014	0.31	0.12	0.79
Middle East and Africa		0.033	0.36	0.14	0.92	0.315	1.99	0.52	7.58	0.024	0.33	0.13	0.86
Southern Europe	Middle East and Africa	0.869	1.06	0.55	2.02	0.513	1.29	0.61	2.73	0.865	0.95	0.50	1.79
Highest Education Attained, Age 20													
Vocational/Technical Training	Compulsory Schooling	0.005	2.46	1.32	4.59	0.609	1.25	0.54	2.89	0.056	1.83	0.98	3.39
Higher Education		0.441	0.79	0.44	1.43	0.275	1.56	0.70	3.48	0.559	0.84	0.46	1.51
Vocational/Technical Training	Higher Education	<0.001	0.32	0.22	0.47	0.341	1.25	0.79	2.00	<0.001	0.46	0.32	0.67
International Socio-economic Index of Occupational Strata													
ISEI Highest 25%	ISEI Lowest 25%	<0.001	3.86	2.29	6.48	0.032	0.51	0.27	0.94	0.018	1.83	1.11	3.02
ISEI Q3		0.002	2.22	1.35	3.65	0.050	0.55	0.30	1.00	0.677	0.90	0.56	1.47
ISEI Q2		0.135	1.46	0.89	2.40	0.267	0.725	0.41	1.28	0.046	0.60	0.37	0.99
ISEI Q2	ISEI Highest 25%	<0.001	0.38	0.23	0.63	0.273	1.43	0.75	2.72	<0.001	0.33	0.20	0.55
ISEI Q3		0.032	0.58	0.35	0.95	0.812	1.09	0.56	2.12	0.006	0.49	0.30	0.81
ISEI Q3	ISEI Q2	0.089	1.52	0.94	2.46	0.378	0.76	0.41	1.41	0.111	1.49	0.91	2.44
Perceptions of Swiss Government Response to COVID-19 pandemic—Effective, Fall 2020													
Effective	Not effective	<0.001	1.97	1.38	2.81	0.103	0.70	0.46	1.08	0.011	1.58	1.11	2.25
Perceptions of Swiss Government Response to COVID-19 pandemic—Agrees to measures, Fall 2020													
Agrees to measures taken	Does not agree	<0.001	2.38	1.61	3.52	0.854	1.05	0.65	1.67	<0.001	2.16	1.46	3.20
Perceptions of Politicians' views of the COVID-19 pandemic—Exaggerated, Fall 2020													
Non-Exaggerated views	Exaggerated views	<0.001	4.704	3.255	6.799	0.309	0.80	0.52	1.23	<0.001	3.72	2.59	5.34
Perceptions of media coverage, Fall 2020													
Fair and Balanced	Not fair or balanced	<0.001	3.152	2.212	4.491	0.373	0.82	0.54	1.26	<0.001	3.56	2.49	5.10
Personal health experiences with COVID-19, April—September 2020													
Only personal health affected	Personal health and close person(s) unaffected	0.074	1.49	0.96	2.31	0.762	1.09	0.63	1.89	0.209	1.32	0.86	2.05
Only health of close person(s) affected		0.873	1.04	0.63	1.73	0.762	1.11	0.58	2.10	0.242	1.36	0.81	2.26
Personal health and health of close person(s) affected		0.091	0.66	0.41	1.07	0.231	1.42	0.80	2.51	0.873	1.04	0.65	1.67
Personal health and health of close person(s) affected	Only personal health affected	0.003	0.44	0.26	0.76	0.416	1.30	0.69	2.45	0.368	0.79	0.47	1.33
Only health of close person(s) affected		0.210	0.70	0.40	1.22	0.969	1.01	0.50	2.04	0.931	1.03	0.59	1.79
Only health of close person(s) affected	Personal health and health of close person(s) affected	0.132	1.58	0.87	2.87	0.494	0.78	0.38	1.59	0.375	1.31	0.73	2.35

Note: Western Europe: Switzerland, Italy, Portugal, Spain, Germany, European Union15 Member States/European Free Trade Association; Southern Europe: Serbia/Montenegro/Kosovo, Bosnia-Herzegovina, Croatia, Macedonia, South & East Europe; Middle East & Africa: North Africa, Sub-Saharan Africa, Near East, Middle & Far East, Turkey; Americas & Other: United States of America, Canada, New Zealand, Australia, Brazil, Other Latin America, Other.

a Close person(s): include family member, partner, or related person.

## Data Availability

Publicly available datasets were analyzed in this study. This data can be found here: Anonymized individual participant data and data dictionaries that underlie the results reported in this article are available to other researchers upon request. Requests including a brief proposal should be sent to DR. As a research infrastructure supported by the SNSF, the *z-proso* study is committed to an open data access policy. Anonymized data, protocols, and other metadata from earlier data collections of the study are generally available to the scientific community. Please contact DR to this purpose.
