# Supplementary material for: Attitudes Toward COVID-19 Vaccination Among Young Adults in Zurich, Switzerland, September 2020
Source: Int J Public Health. 2021 May 6;66:643486. doi: 10.3389/ijph.2021.643486 (PMC8565253; doi:10.3389/ijph.2021.643486)
Supplement: Supplementary file 1 [file DataSheet2.PDF]

Appendix 1

Table 1 – Bivariate logistic regression analyses examining likelihood of getting vaccinated, vaccine hesitancy, and opinion to compulsory vaccination, weighted analyses; ; Zurich Project on the Social Development from Childhood to Adulthood, Zurich, Switzerland, September 2020

		Likelihood of getting vaccinated				Likelihood of being undecided about getting vaccinated				Likely to agree to compulsory vaccine			
	Ref.	*p*	OR	95% CI		*p*	OR	95% CI		*p*	OR	95%CI	
**Sex**													
Female	Male	**0.008**	**0.63**	**0.44**	**0.88**	0.136	1.39	0.90	2.16	**0.003**	**0.59**	**0.42**	**0.83**
**Maternal Country of Birth**													
Southern Europe	Western Europe	**0.008**	**0.53**	**0.33**	**0.85**	0.203	1.43	0.82	2.48	0.254	0.76	0.48	1.22
Sri Lanka		0.451	1.37	0.60	3.12	0.357	0.56	0.16	1.93	**0.043**	**2.43**	**1.03**	**5.76**
Middle East & Africa		**0.012**	**0.50**	**0.29**	**0.86**	0.750	1.11	0.58	2.14	0.418	0.80	0.47	1.37
Americas & Other		**0.040**	**0.45**	**0.21**	**0.96**	0.982	0.99	0.39	2.51	0.144	0.57	0.26	1.21
Southern Europe	Americas & Other	0.711	1.17	0.51	2.72	0.473	1.44	0.53	3.95	0.491	1.34	0.58	3.11
Sri Lanka		**0.042**	**3.06**	**1.04**	**8.95**	0.455	0.57	0.13	2.52	**0.010**	**4.30**	**1.42**	**13.00**
Middle East & Africa		0.816	1.11	0.46	2.68	0.830	1.12	0.39	3.26	0.432	1.42	0.59	3.40
Southern Europe	Sri Lanka	**0.036**	**0.38**	**0.16**	**0.94**	0.155	2.56	0.70	9.31	**0.014**	**0.31**	**0.12**	**0.79**
Middle East & Africa		**0.033**	**0.36**	**0.14**	**0.92**	0.315	1.99	0.52	7.58	**0.024**	**0.33**	**0.13**	**0.86**
Southern Europe	Middle East & Africa	0.869	1.06	0.55	2.02	0.513	1.29	0.61	2.73	0.865	0.95	0.50	1.79
**Highest Education Attained, Age 20**													
Vocational/Technical Training	Compulsory Schooling	**0.005**	**2.46**	**1.32**	**4.59**	0.609	1.25	0.54	2.89	0.056	1.83	0.98	3.39
Higher Education		0.441	0.79	0.44	1.43	0.275	1.56	0.70	3.48	0.559	0.84	0.46	1.51
Vocational/Technical Training	Higher Education	**<0.001**	**0.32**	**0.22**	**0.47**	0.341	1.25	0.79	2.00	**<0.001**	**0.46**	**0.32**	**0.67**
**International Socio-economic Index of Occupational Strata**													
ISEI Highest 25%	ISEI Lowest 25%	**<0.001**	**3.86**	**2.29**	**6.48**	**0.032**	**0.51**	**0.27**	**0.94**	**0.018**	**1.83**	**1.11**	**3.02**
ISEI Q3		**0.002**	**2.22**	**1.35**	**3.65**	**0.050**	**0.55**	**0.30**	**1.00**	0.677	0.90	0.56	1.47
ISEI Q2		0.135	1.46	0.89	2.40	0.267	0.725	0.41	1.28	**0.046**	**0.60**	**0.37**	**0.99**
ISEI Q2	ISEI Highest 25%	**<0.001**	**0.38**	**0.23**	**0.63**	0.273	1.43	0.75	2.72	**<0.001**	**0.33**	**0.20**	**0.55**
ISEI Q3		**0.032**	**0.58**	**0.35**	**0.95**	0.812	1.09	0.56	2.12	**0.006**	**0.49**	**0.30**	**0.81**
ISEI Q3	ISEI Q2	0.089	1.52	0.94	2.46	0.378	0.76	0.41	1.41	0.111	1.49	0.91	2.44
**Perceptions of Swiss Government Response to COVID-19 pandemic – Effective, Fall 2020**													
Effective	Not effective	**<0.001**	**1.97**	**1.38**	**2.81**	0.103	0.70	0.46	1.08	**0.011**	**1.58**	**1.11**	**2.25**
**Perceptions of Swiss Government Response to COVID-19 pandemic - Agrees to measures, Fall 2020**													
Agrees to measures taken	Does not agree	**<0.001**	**2.38**	**1.61**	**3.52**	0.854	1.05	0.65	1.67	**<0.001**	**2.16**	**1.46**	**3.20**
**Perceptions of Politicians' views of the COVID-19 pandemic – Exaggerated, Fall 2020**													
Non-Exaggerated views	Exaggerated views	**<0.001**	**4.704**	**3.255**	**6.799**	0.309	0.80	0.52	1.23	**<0.001**	**3.72**	**2.59**	**5.34**
**Perceptions of media coverage, Fall 2020**													
Fair and Balanced	Not fair or balanced	**<0.001**	**3.152**	**2.212**	**4.491**	0.373	0.82	0.54	1.26	**<0.001**	**3.56**	**2.49**	**5.10**
**Personal health experiences with COVID-19, April – September 2020**													
Only personal health affected	Personal health and close person(s) unaffected	0.074	1.49	0.96	2.31	0.762	1.09	0.63	1.89	0.209	1.32	0.86	2.05
Only health of close person(s) affected		0.873	1.04	0.63	1.73	0.762	1.11	0.58	2.10	0.242	1.36	0.81	2.26
Personal health and health of close person(s) affected		0.091	0.66	0.41	1.07	0.231	1.42	0.80	2.51	0.873	1.04	0.65	1.67
Personal health and health of close person(s) affected	Only personal health affected	**0.003**	**0.44**	**0.26**	**0.76**	0.416	1.30	0.69	2.45	0.368	0.79	0.47	1.33
Only health of close person(s) affected		0.210	0.70	0.40	1.22	0.969	1.01	0.50	2.04	0.931	1.03	0.59	1.79
Only health of close person(s) affected	Personal health and health of close person(s) affected	0.132	1.58	0.87	2.87	0.494	0.78	0.38	1.59	0.375	1.31	0.73	2.35

Note: Western Europe: Switzerland, Italy, Portugal, Spain, Germany, European Union15 Member States/European Free Trade Association; Southern Europe: Serbia/Montenegro/Kosovo, Bosnia-Herzegovina, Croatia, Macedonia, South & East Europe; Middle East & Africa: North Africa, Sub-Saharan Africa, Near East, Middle & Far East, Turkey; Americas & Other: United States of America, Canada, New Zealand, Australia, Brazil, Other Latin America, Other.

$^{\pm}$Close person(s): include family member, partner, or related person
